# When Genetic Diversity Is Low: The Effects of Ploidy Level on Plant Functional Trait Expression in *Spartina* Under Global Change

**DOI:** 10.1002/ece3.71022

**Published:** 2025-03-02

**Authors:** Dirk Granse, Paul Wendt, Sigrid Suchrow, Dieter Hanelt, Jörg Fromm, Morgane Milin, Oscar Lima, Armel Salmon, Malika Aïnouche, Kai Jensen

**Affiliations:** ^1^ Applied Plant Ecology, Institute of Plant Sciences and Microbiology University of Hamburg Hamburg Germany; ^2^ Aquatic Ecophysiology and Phycology, Institute of Plant Sciences and Microbiology University of Hamburg Hamburg Germany; ^3^ Wood Biology, Institute of Wood Science University of Hamburg Hamburg Germany; ^4^ University of Rennes 1, UMR CNRS 6553 Ecobio Rennes Cedex France

**Keywords:** allopolyploidy, climate change, co‐variation in multiple plant traits, hybridization in plants, multivariate trait plasticity index MVPi, plant fitness homeostasis, responses to drought and elevated CO_2_ concentration, *Sporobolus*

## Abstract

Whole genome duplication (WGD or polyploidization) events shape plant evolution, altering ecological responses and plant traits, particularly those related to cell and tissue size. We studied genetic diversity and phenotypic plasticity in *Spartina* populations, focusing on hybrid (*Spartina × townsendii*) and allopolyploid ( *S. anglica* ) cytotypes in Wadden Sea salt marshes. Our results reveal low genetic diversity in both cytotypes and a complex response of plant traits to global change factors (drought, elevated CO_2_ concentration). While WGD increased stomatal length, plasticity varied between cytotypes, with allopolyploids showing higher plasticity, especially under elevated CO_2_. Biomass allocation patterns differed between cytotypes under global change conditions, suggesting distinct effects on ecosystem functioning, such as belowground carbon sequestration and cycling. The allopolyploid's comparatively fewer, larger‐diameter stems may affect aboveground ecosystem functions differently, including sediment trapping and the slowing of tidal currents. Despite similar genetic backgrounds, allopolyploids did not consistently exhibit higher plasticity, challenging previous assumptions. Our findings highlight the complex interplay between hybridization, WGD, phenotypic plasticity, and ecosystem responses to global change, emphasizing the importance of considering polyploidization in understanding plant adaptation and evolutionary dynamics.

## Graphical abstract

Hybridization and whole genome duplication (WGD) in plants can be strong drivers of plant evolution under climate change on relatively short time scales. Genetic divergence was analyzed in *Spartina* populations from the Wadden Sea area approximately 100 years after their introduction. A multivariate plasticity index was used to identify differences between hybrid and allopolyploid responses in plant functional traits to climate change factors, that is, drought and elevated atmospheric CO_2_ concentration. The results indicate low genetic diversity in *Spartina* and ploidy level effects on plant functional traits, especially those considered important for ecosystem functioning.

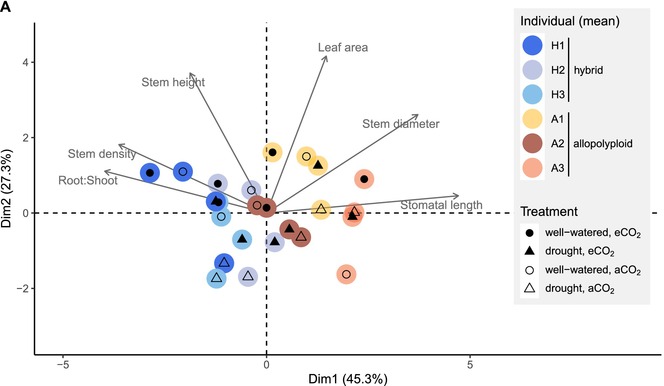

## 1 Introduction

The evolutionary history of plants shows many whole genome duplication events (WGDs), occasionally followed by the emergence of key innovations (Soltis et al. 2014; Soltis and Soltis 2016; Čertner et al. 2017). WGD, or polyploidization, often is broadening ecological tolerances and increasing capacities to respond to changes in the biotic or abiotic environment within two generations (Levin 2019). WGD is accompanied by an increase in cell size, for example, indicated by increases in stomata length (Beaulieu et al. 2008; Hodgson et al. 2010; Masterson 1994; Wet 1954; Speckmann et al. 1965), and decreases in tissue growth rates (Beaulieu et al. 2008; Corneillie et al. 2019). Increases in cell sizes lead to morphological and physiological alterations due to allometric growth effects (reviewed by Leitch and Leitch 2008; Ramsey and Ramsey 2014). Beside genetic effects, such as heterosis and gene dosage compensation (Roose and Gottlieb 1976; Birchler et al. 2001, 2010; Chen 2010; Hollister 2015; Mackay et al. 2021), WGD requires immediate adjustment of inter‐related physiological processes in neopolyploids to establish a “new normal” after WGD (Bomblies 2020), that is, a return to cellular and organismal homeostasis in neopolyploids, accompanied by immediate evolution of functional plant trait expression. These WGD‐mediated adjustments can affect not only the entire plant phenotype, but subsequently also the effects of the phenotype on ecosystem dynamics and functioning (Ainouche et al. 2009; Beest et al. 2012; Guignard et al. 2016). In this study, we ask whether global change factors, such as drought or elevated atmospheric CO_2_ concentrations, interact with WGD‐mediated changes in plant traits and fitness, and how phenotypic plasticity is affected by global change.

Genus *Spartina* (syn.: *Sporobolus*, Poaceae, Peterson et al. 2014; Bortolus et al. 2019) is a well‐suited model system for hybridization and allopolyploidization to address short‐ to medium‐term effects of WGD on plant traits due to its relatively short evolutionary history (< 150 years). The homoploid F_1_‐hybrid (2n = 62) *Spartina × townsendii* H. Groves & J. Groves (syn.: *Sporobolus × townsendii* P.M.Peterson & Saarela), first collected in 1870, formed in southern England from parental *Spartina alterniflora* Loiseleur (syn.: *Sporobolus alterniflorus* P.M.Peterson & Saarela, 2n = 62) and *Spartina maritima* (Curtis) Fernald (syn.: *Sporobolus maritimus* P.M.Peterson & Saarela, 2n = 60). Following whole genome duplication, the allopolyploid (2n = 124) descendant *Spartina anglica* C.E. Hubbard (syn.: *Sporobolus anglicus* P.M.Peterson & Saarela) was recorded at the same location (Marchant 1967, 1968). Comparative chromosome numbers among various *Spartina* species led Marchant (1968) to suggest x = 10 as a base chromosome number in *Spartina*, and thus hexaploidy for *S. alterniflora* , *S. maritima* and their hybrid *S. × townsendii* and (allo)dodecaploidy for *S. anglica* . While *S. × townsendii* is almost infertile (Marchant 1968; Granse, Romeiro Motta, et al. 2022), *S. anglica* varies largely in seed set and seed germination success (Marchant 1968; Marks and Truscott 1985). *S. × townsendii* and *S. anglica* were deliberately introduced in salt marshes around the globe since the 1920s. The abiotic dynamics in salt marsh habitats, mainly tidal salt water regimes (Gray et al. 1991), may have exposed *Spartina* development to selective constraints over the last century, which was recently demonstrated in parental *S. alterniflora* (Hao et al. 2024). However, little is known about the genetic diversity of these *Spartina* hybrids in salt marshes from microhabitats to continent scales and particularly in view of genetic bottlenecks that occurred during the formation of both the hybrid *S. × townsendii* and its allopolyploid descendant, *S. anglica* (Baumel et al. 2001), suggesting low genetic divergence within these populations. Low genetic diversity can weaken the effectiveness of selective evolution and highlights the need for both adaptation to global change through phenotypic plasticity and harnessing the benefits of hybridization and WGD.

*S. × townsendii* and *S. anglica* , in the following termed “hybrid” and “allopolyploid” *Spartina* cytotypes, both perform C_4_ photosynthesis (Long et al. 1975). The cytotypes differ in plant traits, including stem density, stem diameter, leaf area, and the angle between the stem and leaf (Marchant 1967; Partridge 1987; Wong et al. 2018). Due to greater morphological variation (Thompson et al. 1991b) and gene expression variation (Ainouche et al. 2012), the allopolyploid *S. anglica* was hypothesized to benefit under changing environmental conditions from greater phenotypic plasticity than its parental species.

The principal objectives of our study were to investigate genetic diversity in *Spartina* populations from the geographic range of the Wadden Sea area and in microhabitats, assuming that different geographic regions and microhabitats harbor different genotypes due to variation in abiotic parameters (e.g., flooding regime, salinity) which may act as selective evolutionary force (cf. Bockelmann et al. 2003; Rouger and Jump 2015; Castillo et al. 2018). Prior to a greenhouse experiment, we identified specific *Spartina* genotypes by microsatellite marker analysis. In a greenhouse experiment, the interacting effects of environmental factors and WGD were studied to assess the extent to which WGD controls plant trait expression and plasticity under different environmental conditions, that is, drought versus well‐watered and elevated vs. ambient atmospheric CO_2_ concentrations. Drought is one of the most limiting abiotic factors of terrestrial plant productivity (Boyer 1982; Pavlíková et al. 2017), while elevated CO_2_ concentrations can have significant effects on plant physiology (e.g., Rogers et al. 1994; Zheng et al. 2018) and primary productivity (Ueyama et al. 2020). In C_4_ species, elevated atmospheric CO_2_ concentrations improve plant water relations by delaying and ameliorating drought stress, which results from the interaction of water stress with reduced stomatal conductance at elevated CO_2_ concentrations, thereby indirectly enhancing photosynthesis, growth, and yield (Wand et al. 1999; Ainsworth and Long 2005; Ainsworth and Rogers 2007; Leakey et al. 2009; Vanuytrecht et al. 2012).

In the greenhouse experiment, we focused on phenotypic plasticity expressed as treatment‐induced variation in stomatal length and five plant functional traits with a high eco‐evolutionary potential (stem diameter, stem density, stem height, root‐to‐shoot ratio, leaf area) and, furthermore, in plant biomass as a proxy for fitness (cf. Vahsen, Blum, et al. 2023; Vahsen, Kleiner, et al. 2023). This multivariate phenotypic plasticity approach considers that various traits can change interactively at the same time in response to environmental factors (Nielsen and Papaj 2022). We used a multivariate plasticity index (MVPi; Pennacchi et al. 2021) to assess phenotypic plasticity, which is commonly defined as the ability of specific genotypes to show different phenotypes in response to different environmental conditions (Pigliucci et al. 2006). In this study, we address the following hypotheses:

1. The *Spartina* hybrid and its allopolyploid descendant show low genetic diversity in Wadden Sea populations, approximately 100 years after their introduction to the study area. This was tested by means of microsatellite marker analysis.
2. Plant functional trait plasticity measured as the multivariate plasticity index increases with WGD and thus is higher in the allopolyploid descendant compared to the hybrid.
3. WGD leads to increased plant fitness in response to elevated CO_2_ levels and drought conditions. Plant fitness is therefore higher in the allopolyploid descendant compared to the hybrid under the combined treatment of elevated CO_2_ and drought.

## 2 Materials and Methods

### 2.1 Brief Taxon Description and Field Sampling

**FIGURE 1:**
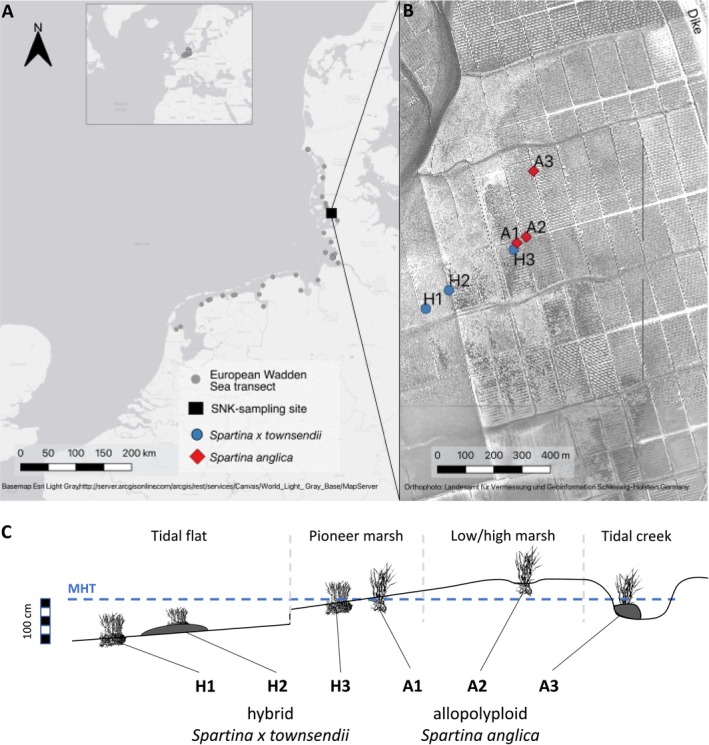
Sampling positions of *Spartina* along a European Wadden Sea transect (A) for microsatellite marker analysis (simple‐sequence repeats: SSR). Sampling site (B) at Sönke‐Nissen‐Koog (SNK), Schleswig‐Holstein, Germany, harboring donor individuals (hybrid: H1–H3; allopolyploid: A1–A3) for the greenhouse experiment. Schematic view (C) of sampling positions of *Spartina* donor individuals along an elevational gradient (tidal flat, pioneer marsh, low/high marsh, tidal creek) in relation to mean high tide (MHT; Table A1).

*S. × townsendii* and *S. anglica* thrive along the eastern part of the European Wadden Sea (Figure 1) in sympatric populations in salt marshes, along the elevational gradient mainly in the pioneer and low marsh zone (Granse, Romeiro Motta, et al. 2022). Fresh leaves from *Spartina* plants were collected from July to November 2017 at 33 locations along an European Wadden Sea transect from Den Helder (Netherlands) to Skallingen (Denmark; Figure 1A; as described in Granse, Romeiro Motta, et al. 2022) and at 46 positions in different microhabitats (tidal flat, pioneer marsh, low and high marsh, tidal creek) of two salt marsh sites (N53°58′35″ E8°53′0″ and N54°38′0″ E8°50′18″) in Schleswig‐Holstein, Germany.

For the greenhouse experiment, fragments of three hybrid *S. × townsendii* (H1–H3; Table A1) and three allopolyploid *S. anglica* (A1–A3) donor individuals were sampled at Sönke‐Nissen‐Koog (Schleswig‐Holstein, Germany; Figure 1B) in August 2017. The sampling positions varied in elevation and microhabitat (Figure 1C; cf. Granse, Titschack, et al. 2022). The stem density was determined in an area of 20 *×* 20 cm, and the height and stem diameter at the third leaf were measured on three of the tallest stems using a caliper and a ruler to record plant traits at field sites as a reference for identifying legacy effects (Table A1). Elevation was measured using a Trimble laser leveling device (LL500 combined with HL700 receiver by Trimble, CA, USA). Fragments of the sampled plants were potted in native soil and kept in the greenhouse under freshwater conditions and weekly KNP fertilization (1% v/v) until preparation for the greenhouse experiment.

### 2.2 Cytotype and Genotype Analyses, Genetic Diversity

The cytotypes of 79 *Spartina* plants were determined using a DAPI staining protocol (standard = *Pisum sativum* ) for flow‐cytometer analysis (Partec GmbH, Münster, Germany; see Granse, Romeiro Motta, et al. 2022). Out of the 79 individual *Spartina* plants, including the donor individuals tested in the greenhouse experiment (H1–H3, A1–A3; Figure 1B,C), seven plants were determined as hybrid (five from microhabitats) and 72 as allopolyploid (40 from microhabitats) cytotype.

DNA was extracted from ground plant material using a Nucleospin plant 2 MINI kit (Macherey‐Nagel, Düren, Germany) following the manufacturer's protocol with minor modifications, that is, an additional centrifugation step (11,000 g for 1 min) was applied after cell lysis and by using 650 μL of PW2 for washing the silica membrane. Samples with high debris content were rectified with 200 μL PL1 and 5 μL RNase. Normalized DNA was analyzed by using FAM‐labeled primers of eight SSR markers and by applying a standardized PCR procedure (Tables A3 and A4). The PCR products were analyzed by means of a capillary electrophoresis sequencer and by using GeneScanTM‐500 LIZ as a size standard. The microsatellite marker alleles were determined from the resulting electropherograms using GeneMapper v4.1 (Applied Biosystems, California, USA) and visually identified binning classes.

Genotypes of samples were determined using eight microsatellite markers (MS02, MS07, MS13–MS18) that were amplified with primers designed in conserved regions from genomic sequences (simple‐sequence repeats, SSR; Table A2) of parental *S. maritima* (Castillo et al. 2018) suitable for genotyping in related species. The set of SSR markers included sequence repeats with motif lengths ranging from 4 to 6 bp. To avoid overestimation of SSR marker polymorphisms caused by additional insertion–deletion, two alleles from SSR marker MS15 and MS17 were excluded, respectively, because the fragment sizes were conspicuously lower than the fragment size range of most frequent alleles. All data on SSR marker are accessible in a public data repository (Granse et al. 2024b).

### 2.3 Greenhouse Experiment

**FIGURE 2:**
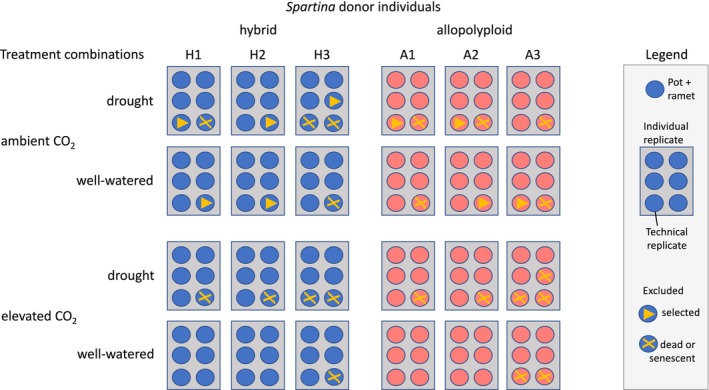
Experimental design testing six *Spartina* donor individuals (hybrid: H1–H3; allopolyploid: A1–A3) under drought (vs. well‐watered) and elevated CO_2_ (950 ppm vs. ambient CO_2_) level treatment combinations. Individual replicates consist of six ramets (pots = technical replicates; *n* = 144). Surviving ramets at the end of the experiment were included into the statistical evaluation (*n* = 114). Excluded from analysis: Dead or senescent plants (*n* = 21), and randomly selected plants (*n* = 9) in preparation for use in subsequent experiments.

In April 2018, ramets showing a first leaf with a length of 5 cm and a rhizome of 10 cm in length were harvested from the six *Spartina* donor individuals (Figure 1C). Branches of the rhizome and fine roots were removed. This was done to ensure equal establishment conditions of ramets similar in size. Seedlings could not be used because *S. × townsendii* is sterile. Six ramets in pots (technical replicates) per individual replicate and per treatment combination were used in a full‐factorial experimental design (Figure 2) under two levels of water availability (drought: soil water content < 10% v/v, control: well‐watered > 30% v/v) and two atmospheric CO_2_ concentrations (elevated CO_2_: 950 ppm, ambient CO_2_: 400 ppm). Each of the 144 ramets was grown in a 2 L pot with an autoclaved soil mixture (1:1) of sand (0.13–0.36 mm grain size fraction) and commercial compost. The six replicate pots per treatment combination were kept in a tray during the experimental phase from May to October 2018. The pots in a tray as well as the tray positions were rotated weekly. The plants were weekly fertilized with commercial Hakaphos Blau (15‐10‐15) NPK fertilizer solution (1% v/v; 50 mL per pot). Overall, 114 out of the 144 pots remained for the analyses at the end of the experiment (for details see Figure 2).

Water availability treatments were realized by providing 50 mL freshwater (drought treatment) to each pot per week, while the control pots (well‐watered treatment) received additional freshwater to realize a volumetric soil water content of > 30%. The volumetric water contents (% v/v) of the pots were weekly monitored using a TDR meter (Delta‐T Devices, Cambridge, UK). The CO_2_ enrichment of the atmosphere was conducted in the greenhouse at the Institute of Wood Science, University of Hamburg, Germany, in two climate chambers (ambient and elevated level; according to Tom‐Dery et al. 2019) under greenhouse daylight conditions (day/night air temperature 27°C/22°C; 70%–80% relative air humidity; length of photo‐period as according to northern Germany region from May to October).

### 2.4 Plant Stomatal Length

The third leaf of the tallest plant of an individual replicate was cut off after the end of the experimental treatment period (May to October) and stored in 70% ethanol. The leaf samples were fixed in microtubule stabilization buffer (MSB) including 2% paraformaldehyde (PFA) for 3 h. The fixed samples were dewatered in an ascending alcohol series (30%–100% ethanol) and incubated in an ethanol LR‐White Resin (London Resin Company, Reading, UK) mixture in a ratio of 1:1 for 2 h. Afterwards, the samples were transferred into pure LR‐White Resin for 12 h and embedded in gelatin capsules. The polymerization was carried out in an incubator at 50°C for 36 h. The polymerized samples were cut along the inner surface of the leaf groove into 1 μm thick half‐thin sections using an ultramicrotome (Ultracut E, Reichert Jung, Vienna) with a glass knife (for an illustration see Figure A2).

**FIGURE 3:**
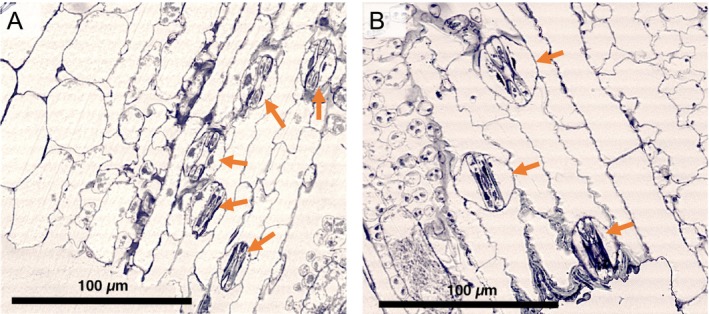
Plant stomata in hybrid *S. × townsendii* (A) and allopolyploid *S. anglica* (B). The orange arrows point to stomata with two dumbbell‐shaped guard cells flanked by two lateral subsidiary cells, respectively.

**FIGURE A1:**
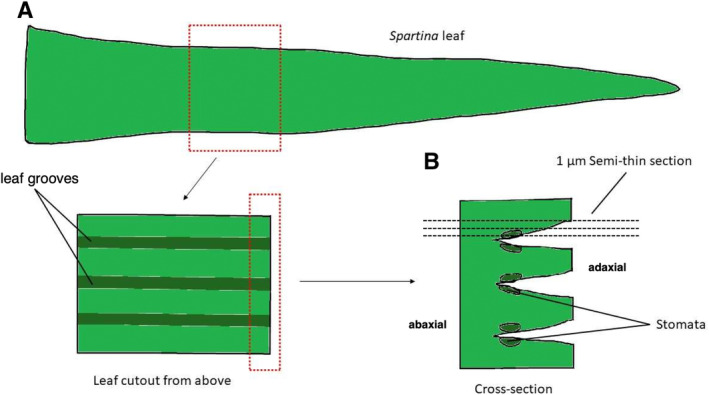
Preparation of a *Spartina* leaf for microtomic cut. Enlarged section of the leaf (A) from above with the characteristic leaf grooves. Enlarged cross section (B) of the *Spartina* leaf with the stomata located at the bottom of the leaf groove. The dashed black lines show the position where the cuts were made.

**FIGURE A2:**
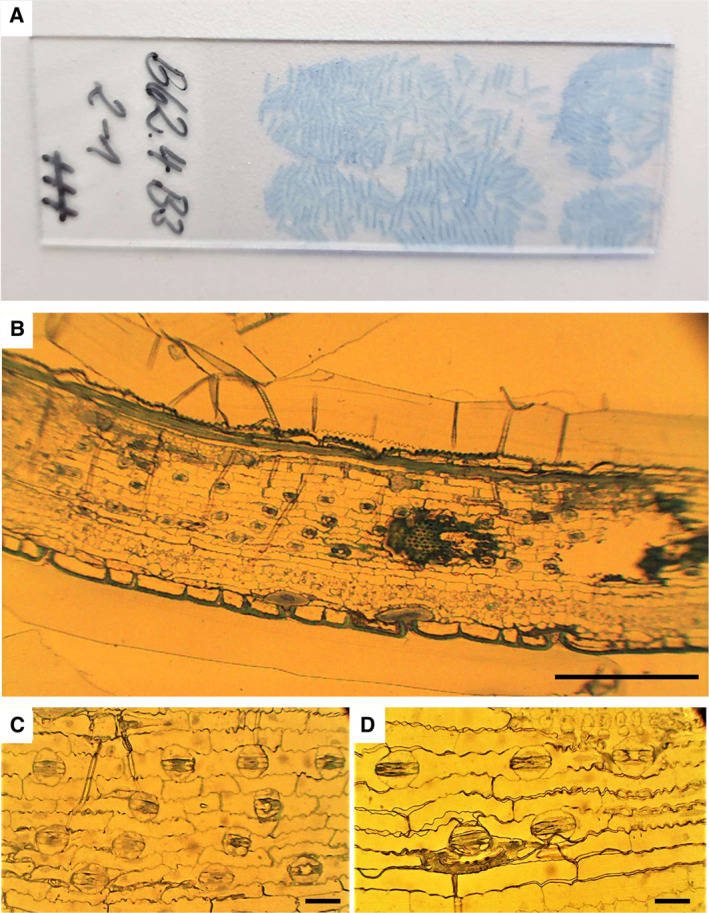
Microscope slide (A) with the 1 μm thick semi‐thin microtomic cuts. Semi‐thin cut (B) at 10× magnification. Semi‐thin cut (C) at 40× magnification. Both (B, C) show stomata of a homoploid *Spartina* F_1_‐hybrid individual. Semi‐thin microtomic cut (D) at 40× magnification displaying the stomata of an allopolyploid *Spartina* individual. Bars: 120 μm (B) and 25 μm (C, D).

The cuts were placed on the slide (see Figure A1A) and stained with 0.05% toluidine blue in 50 mM MSB. The evaluation of stomata (Figure 3) was carried out under a transmission light microscope (Olympus BH‐2, Olympus Optical CO., LTD, Japan) with an attached camera (Bresser MikroCam SP 5.0, Germany). The stomatal length was measured at six or more stomata per replicate with the camera software Bresser CamLabLite 2.0 (*n* = 550; Figure A1C,D).

### 2.5 Biomass, Stem, and Leaf Related Traits

At the end of the experiment, stems per pot were counted and reported as stem density. Stem diameter, leaf width, and leaf length were measured on the third leaf using a caliper at three of the tallest vital stems per pot (reported as mean‐value per pot). The leaf area was calculated as leaf width *×* leaf length (reported as the mean of three stems), by means of the linear regression model of

(1) $$\text{area}=86+\left(0.63224\times \text{width}\times \text{length}\right)$$

The model was fitted (*R* ^2^ ≥ 0.98) with leaves from 10 hybrid and allopolyploid *Spartina* samples, respectively, which were grown under ambient greenhouse conditions and by measuring the leaf area using a portable leaf area meter (AM300, ADC BioScience Ltd., UK).

The plant dry biomass was measured after oven‐drying below‐ and aboveground plant material at 65°C for 48 h. The relative decrease in biomass under drought treatment was calculated based on the biomass (100%) accumulated under well‐watered condition. All data on the greenhouse experiment are accessible in a public data repository (Granse et al. 2024a).

### 2.6 Multivariate Plasticity Index

The multivariate plasticity index (MVPi) is reported according to Pennacchi et al. (2021) by using the scores of a principle component analysis (PCA) which forms a “trait‐scape” on a selection of plant functional traits (stomatal length, root:shoot ratio, leaf area, stem height, stem diameter, and stem density) to calculate the Euclidean distance for each of the *Spartina* individuals (H1–H3, A1–A3) from PCA coordinates of their technical replicates.

(2) $${\mathrm{ED}}_{X‐Y}=\sqrt{\left[{\left({\mathrm{Amb}}_{{\mathrm{CV}}_{1}}‐{\mathrm{Tr}}_{{\mathrm{CV}}_{1}}\right)}^{2}+{\left({\mathrm{Amb}}_{{\mathrm{CV}}_{2}}‐{\mathrm{Tr}}_{{\mathrm{CV}}_{2}}\right)}^{2}+{\left({\mathrm{Amb}}_{{\mathrm{CV}}_{k}}‐{\mathrm{Tr}}_{{\mathrm{CV}}_{k}}\right)}^{2}\right]}$$

ED is the Euclidean distance between the technical replicates (*X*) of the ambient (Amb) environment and the replicates (*Y*) from the treatment (Tr) environment of the same *Spartina* individual. CV_1_ –CV_k_ are the scores (coordinates) of the six (*k*) dimensions from the PCA analysis, where *k* is equal to the number of evaluated plant traits.

The multivariate plasticity index (MVPi) was calculated as the mean of the Euclidean distances for each of the *Spartina* individuals.

(3) $$\begin{array}{cc} \text{MVPi}\;=\; & ({\mathrm{ED}}_{1-1}+{\mathrm{ED}}_{1-2}+\ldots +{\mathrm{ED}}_{1-m}+{\mathrm{ED}}_{2-1}+\ldots \\ & +{\mathrm{ED}}_{2-n}+{\mathrm{ED}}_{n-1}+\ldots +{\mathrm{ED}}_{n-m})/\left(n.m\right) \end{array}$$

where ED is the Euclidean distance (Equation 2) from *n* individuals of the ambient environment (*X*) and *m* individuals under the treatment (*Y*). For the full method description, see Pennacchi et al. (2021).

The plant trait variation index is a measure of the similarity in traits expression of individuals or groups in relation to the overall mean proxied by the center of the ordination. This index is reported as Euclidean distance per *Spartina* individual and was calculated from the PCA scores (coordinates) of all PCA dimensions in the ambient environment (well‐watered, ambient CO_2_ level) in relation to the centroid of the PCA scores (initial point).

### 2.7 Statistics

A minimum spanning networks diagram for *Spartina* genotypes was created using the function poppr.msn() from the R package poppr (Kamvar et al. 2015) and based on a dissimilarity matrix calculated with the function diss.dist().

The multivariate plasticity indices for the treatment combinations (standardized Euclidean distance and MVPi) of the *Spartina* individuals (H1–H3, A1–A3) were compared using a one‐way ANOVA (aov) followed by Tukey‐HSD post hoc analyses. Multivariate analysis was conducted by imputing missing values with the function imputePCA() from the R package missMDA (Josse and Husson 2016) as a preliminary step before performing the function PCA() from the package FactoMineR (Lê et al. 2008) on the completed dataset. Ordination diagrams were created by using the function fviz_pca_biplot() from the R package factoextra (Kassambara and Mundt 2020).

The plant traits of stomatal length, biomass, root‐to‐shoot ratio, leaf area, stem height, stem diameter, and stem density are reported and evaluated as mean values calculated from the surviving plants per individual replicate and treatment combination.

The response of the cytotypes to drought and elevated CO_2_ level was evaluated by means of a three‐way ANOVA based upon the full‐factorial experimental design. The ANOVAs were conducted by using cytotype (hybrid, allopolyploid), _treat_H_2_O (well‐watered, drought), and _treat_CO_2_ (ambient, elevated CO_2_ level) as fixed factors: Response ∼ Cytotype × (_treat_H_2_O × _treat_CO_2_). To achieve data normality, the response data was transformed by means of the BoxCox method ((response^λ^—1)/λ). Normality was evaluated using Shapiro–Wilk tests (shapiro.test) and homogeneity of variance across groups using Levine tests (leveneTest). The ANOVA (aov) results were post hoc analyzed by means of Tukey‐HSD tests.

All statistical methods were implemented in R version 4.2.2 (R Core Team 2022).

## 3 Results

### 3.1 Genetic Diversity

**FIGURE 4:**
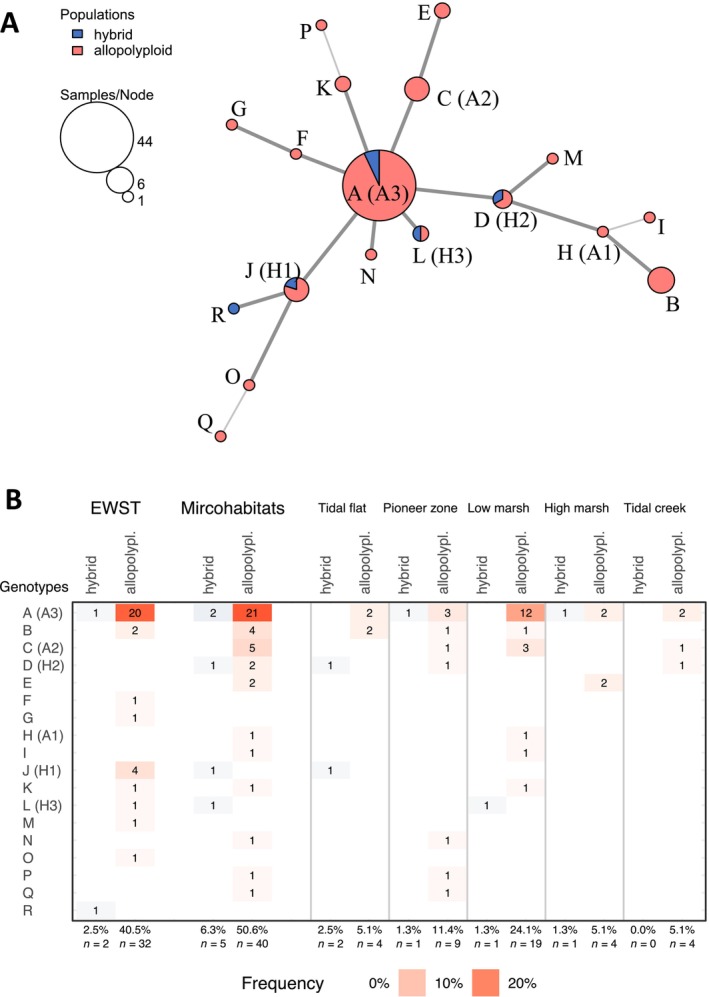
Minimum spanning networks (A) and frequency (B) of multilocus genotypes (*A*–*R*) of 79 *Spartina* samples (hybrid: *n* = 7; allopolyploid: *n* = 72) from the European Wadden Sea transect (EWST) and microhabitats (tidal flat, pioneer zone, low marsh, high marsh, tidal creek), based on eight SSR polymorphic loci. The genotypes of the individuals (hybrid: H1–H3; allopolyploid: A1–A3) are labeled. The table in (B) represents frequency (color) and count (*n*) for genotypes.

Overall, 18 multilocus genotypes were found with a frequency of 55.7% in the most prominent genotype (*A*; *n* = 79; Figure 4A). The genotype *A* was represented by 42.9% (3/7) of hybrid and 56.9% (41/72) of allopolyploid samples (Table A5). In contrast, 17 multilocus genotypes (*B*–*R*) showed relatively low frequencies (min: 1.3% = one sample; max: 7.6% = six samples) due to low‐frequency alleles (rare alleles). Eight genotypes (*B*–*I*) shared all 16 SSR marker alleles with genotype *A*, but showed up to four additional alleles. Between one and three alleles were missing in the most diverging nine genotypes (*J*–*R*) compared with genotype *A*. In consequence, this would indicate low genetic divergence among investigated *Spartina* samples as well as between hybrid and allopolyploid *Spartina* cytotypes.

Between two and five different alleles per locus were found at the eight surveyed microsatellite loci, showing an overall 16 alleles in the *Spartina* samples from European Wadden Sea marshes (Table 1). The allelic number per SSR locus ranged from 1 to 5, where 3 out of 8 SSR markers (MS2, MS14, MS18) exhibited the lowest polymorphism. There was no indication that alleles or combinations of alleles (genotypes) were specific to any geographic region of the Wadden Sea or to any of the microhabitats (Figure 4B). In consequence, the diversity of alleles and genotypes did not differ among the sampled Wadden Sea sites from the Netherlands to Denmark, as well as among the microhabitats.

**TABLE 1:** Genotype frequency per SSR marker in 79 *Spartina* samples from the European Wadden Sea, including the hybrid (individuals: H1–H3) and its allopolyploid descendant (individuals: A1–A3). The number (*n*) of individuals is given in brackets for the corresponding genotypes.

SSR marker	Genotypes (fragment sizes in bp)	Frequency (%, *n*)			*Spartina* individuals	
		Hybrid	Allopolyploid	Total		
		(*n* = 7)	(*n* = 72)	(*n* = 79)	Hybrid	Allopolyploid
MS02	268	100.0 (7)	87.5 (63)	88.6	H1, H2, H3	A2, A3
MS02	257/268	0.0 (0)	12.5 (9)	11.4		A1
MS07	257	14.3 (1)	1.4 (1)	2.5	H3	
MS07	257/273	85.7 (6)	98.6 (71)	97.5	H1, H2	A1, A2, A3
MS13	248	0.0 (0)	1.4 (1)	1.3		
MS13	248/264	85.7 (6)	93.1 (67)	92.4	H1, H3	A2, A3
MS13	238/248/264	14.3 (1)	5.6 (4)	6.3	H2	A1
MS14	271	100.0 (7)	95.8 (69)	96.2	H1, H2, H3	A1, A2, A3
MS14	260/271	0.0 (0)	2.8 (2)	2.5		
MS14	268/271	0.0 (0)	1.4 (1)	1.3		
MS15	250	14.3 (1)	0.0 (0)	1.3		
MS15	223/250/261	14.3 (1)	6.9 (5)	7.6	H1	
MS15	223/242/250/258	0.0 (0)	1.4 (1)	1.3		
MS15	223/250/258/261	71.4 (5)	91.7 (66)	89.9	H2, H3	A1, A2, A3
MS16	204/251/260	100.0 (7)	100.0 (72)	100.0	H1, H2, H3	A1, A2, A3
MS17	264	0.0 (0)	4.2 (3)	3.8		
MS17	264/273	100.0 (7)	95.8 (69)	96.2	H1, H2, H3	A1, A2, A3
MS18	260	100.0 (7)	84.7 (61)	86.1	H1, H2, H3	A1, A3
MS18	245/260	0.0 (0)	1.4 (1)	1.3		
MS18	245/288	0.0 (0)	1.4 (1)	1.3		
MS18	250/260	0.0 (0)	9.7 (7)	8.9		A2
MS18	260/280	0.0 (0)	2.8 (2)	2.5		

### 3.2 WGD Effects on Plasticity in Functional Traits

**FIGURE 5:**
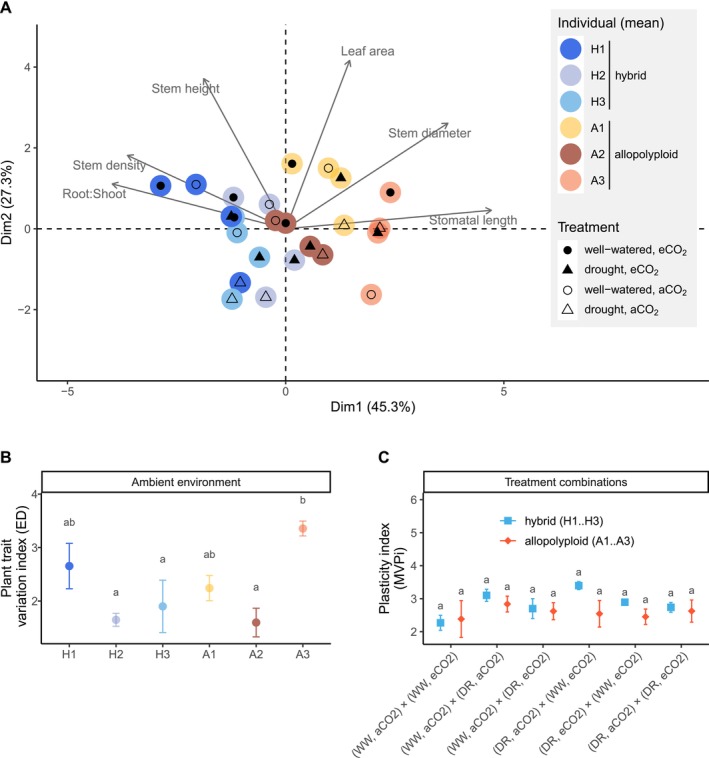
Multivariate trait‐scape (A; ordination plot) for PCA analysis of functional plant traits (root‐to‐shoot ratio, stem density, stem height, leaf area, stem diameter, stomatal length) of three hybrid (H1–H3) and three allopolyploid (A1–A3) *Spartina* individuals in response to treatment combinations of drought (DR) versus well‐watered (WW) × elevated (eCO_2_) versus ambient (aCO_2_) level. Plant trait variation index (B) of individuals for all PCA dimensions related to the PCA centroid (Euclidean distance ED), and multivariate plasticity index MVPi (C) for ploidy levels (hybrid, allopolyploid) under treatment combinations. The letter‐codes (a, b) in (B) indicate differences between plant individuals (ANOVA followed by Tukey HSD post hoc tests, *F* = 4.4, *p* < 0.05, df = 5; Means and error whiskers = standard error) but no differences in (C) between ploidy levels (ANOVA, *F* = 2.7, *p* = 0.11, df = 1). More details are given in the Figures A3, A4, A5, A6.

The first two dimensions of the multivariate trait‐scape explained 72.6% of the variations of *Spartina* individuals in functional plant traits in response to treatment combinations of drought vs. well‐watered × elevated vs. ambient CO_2_ level (Figure 5A). Stomatal length, root‐to‐shoot ratio, stem diameter, and stem density were highly correlated with the first trait‐scape dimension which represented the ploidy level (Figure A4, Dim1). Leaf area and stem height strongly correlated with plant fitness (proxied by biomass) in the second trait‐scape dimension (Figure A4, Dim2).

Individual allopolyploid plants showed differences in plant trait variation in the ambient environment (*p* = 0.03, Figure 5B) while trait variation did not differ between hybrid individuals. Although no differences in MVPi were observed between the hybrid and allopolyploid *Spartina* cytotypes under the treatment combinations tested (*p* = 0.1, Figure 5C), the allopolyploid individuals differed in MVPi, particularly under the different CO_2_ level environments (*p* < 0.02, Figure A6). This would mean that in response to changes in CO_2_ level, allopolyploid individuals tend to show higher phenotypic plasticity than hybrid individuals.

### 3.3 Impact of WGD on Plant Traits, Biomass, and Biomass Allocation

**FIGURE 6:**
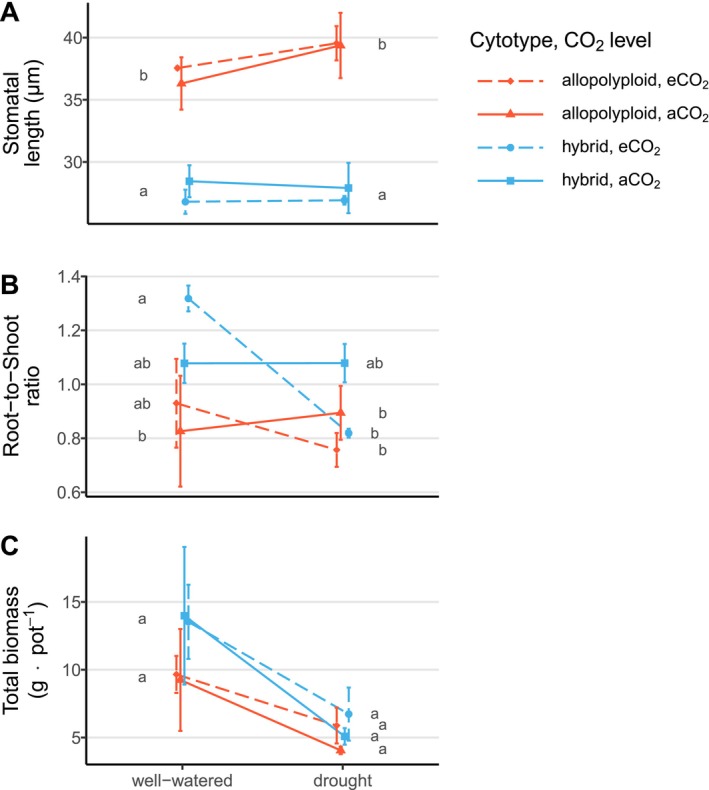
Interaction plots of stomatal length (A), root‐to‐shoot ratio (B), and total biomass (C) response of *Spartina* cytotypes (hybrid, allopolyploid) to water availability (well‐watered, drought) versus CO_2_ level (ambient, elevated) treatments. The letter‐codes (a, b) indicate differences between cytotype, water availability and CO_2_ level treatments (ANOVA followed by Tukey HSD post hoc tests, *p* < 0.05, df = 1; (A): Cytotype effects *p* < 0.01; (B): Drought × CO_2_ level effects *p* < 0.05; (C): Drought effects *p* < 0.01; A detailed statistic is reported in Table A6; Means and error whiskers = standard error).

The stomatal length increased after WGD in *Spartina*, that is, hybrid *S. × townsendii* vs. allopolyploid *S. anglica* , from 27.5 μm ± 2.1 SD (hybrid, mean) to 38.2 μm ± 3.0 SD (allopolyploid, mean; *p* < 0.001, Figure 6A; Table A6), however, the cytotypes showed no differences in stomatal length among treatments.

The root‐to‐shoot ratio was highest in the hybrid under ambient water availability and elevated CO_2_ levels and decreased under drought and elevated CO_2_ levels (Figure 6B; *p* < 0.05; Table A6), while the root‐to‐shoot ratio of the allopolyploid was not affected by drought and elevated CO_2_ levels (Figure A8).

The hybrid and allopolyploid *Spartina* cytotypes showed the highest biomass under well‐watered conditions and the lowest biomass under drought (Figure 6C, *p* < 0.01, Table A6). The biomass was slightly higher in the hybrid under well‐watered condition (mean: 13.8 g ± 6.3 SD) than in the allopolyploid (mean: 9.5 g ± 4.4 SD), whereas biomass of both cytotypes decreased towards lower and rather similar values under drought (mean: 5.9 g ± 2.4 SD and 5.0 g ± 1.8 SD; Figure 6C). This decrease in biomass due to drought, although not significant (*p* ≥ 0.05, Table A6), was more pronounced in the hybrid (−57%) than in the allopolyploid (−47%) and with a higher difference in biomass decrease (hybrid: −52%; allopolyploid: −36%) if comparing control (ambient CO_2_ level, well‐watered) with the full treatment (elevated CO_2_ level, drought).

## 4 Discussion

### 4.1 Genetic Diversity

The analysis of genetic diversity based on eight microsatellite loci revealed low inter‐individual genetic diversity both among hybrid (*S. × townsendii*) individuals and among allopolyploid ( *S. anglica* ) individuals. A similar trend towards low genetic diversity was observed among the sampled Wadden Sea sites and the different microhabitats. This supports our first hypothesis that the *Spartina* hybrid and its allopolyploid descendant show low genetic diversity in Wadden Sea populations.

The results of this study agree with findings from previous molecular investigations in European *Spartina* populations (Raybould et al. 1991; Baumel et al. 2001; Ainouche et al. 2004) using different genetic markers, where one “major” multilocus genotype was encountered. Our results are also consistent with a genetic bottleneck occurring during the formation of the hybrid and the allopolyploid, and the predominant vegetative propagation after their formation. We also see that the hybrid and its allopolyploid descendant have very similar allelic and genotypic composition at these loci. This agrees with the recent origin of the hybrid and the allopolyploid, where parental genome additivity (from *S. alterniflora* and *S. maritima* ) is being observed in both *S. × townsendii* and *S. anglica* (Salmon et al. 2005; Ainouche et al. 2012).

### 4.2 WGD Effects on Plasticity in Functional Traits

A greater stomatal length in the allopolyploid in comparison to the hybrid (Figure 6A) is consistent with the observation that cell size generally increases after WGD (cf. Beaulieu et al. 2008; Roddy et al. 2020; Doyle and Coate 2019). However, the stomatal length in hybrid and allopolyploid *Spartina* cytotypes did not change in response to the experimental global change factors, neither under drought nor under elevated CO_2_ levels.

Stomatal length, root‐to‐shoot ratio, stem diameter, and stem density were associated with the first dimension of the multivariate trait‐scape and explained at least 45.3% of variation (Figure 5A, Figure A4). Although these plant traits are highly affected by WGD (e.g., Marchant 1967; Wong et al. 2018; Granse, Romeiro Motta, et al. 2022) and trait variation was particularly high between allopolyploid individuals (Figure 5B), no differences in plasticity were observed between the cytotypes (Figure 5C). This observation did not support our second hypothesis that plant functional trait plasticity increases with WGD.

Earlier studies of Stebbins (1971) and Levin (1983) demonstrated that allopolyploids can show a higher phenotypic plasticity due to increased genomic redundancy. However, the results of these studies of a higher WGD‐mediated trait plasticity are contrasting to recent observations in natural allopolyploids of wild strawberry (*Fragaria*; Wei et al. 2019) or *Arabidopsis* (Kornstad et al. 2022; Shimizu‐Inatsugi et al. 2023), but similar to the observations in newly synthesized *Arabidopsis kamchatica* allopolyploids (Shimizu‐Inatsugi et al. 2023). In those studies, allopolyploids were compared to their parents, but not directly to the F_1_‐hybrid progenitor, as addressed in our study. Based on a novel multivariate approach, our data did not confirm that WGD leads to a generally higher phenotypic plasticity.

**FIGURE A3:**
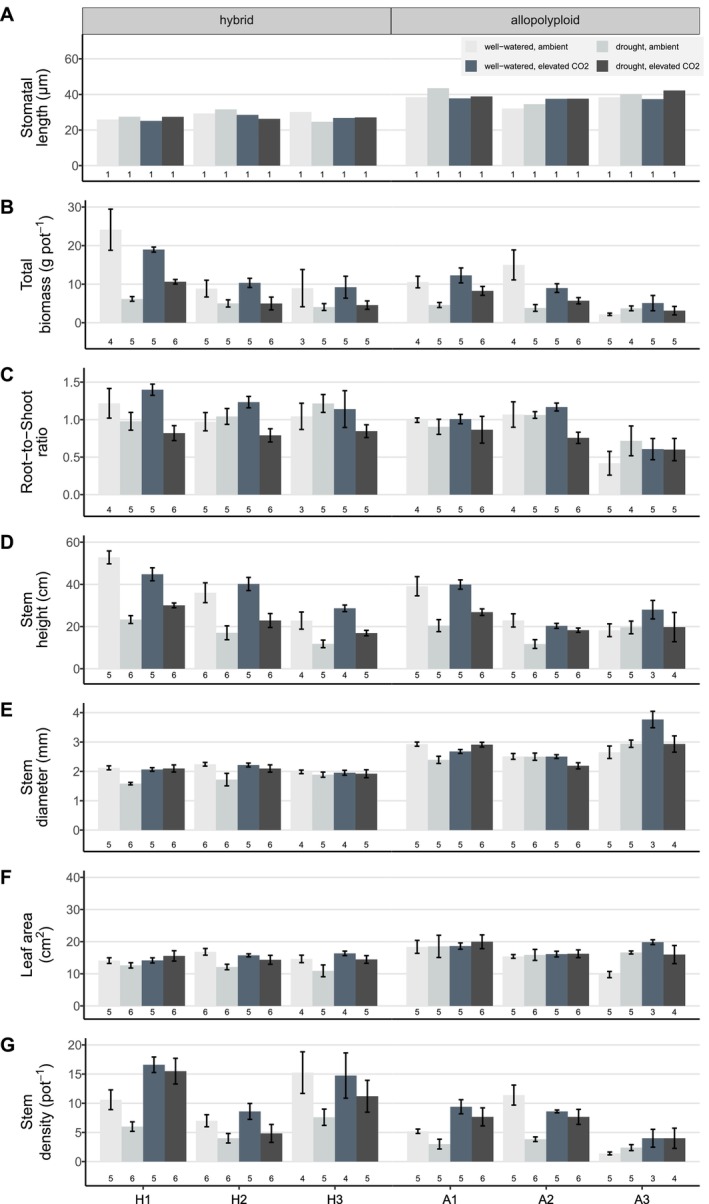
Stomatal length (A), biomass (B), root‐to‐shoot ratio (C), stem height (D), stem diameter (E), leaf area (F), and stem density (G) of hybrid (H1–H3) and allopolyploid (A1–A3) *Spartina* individuals. The numbers below the bars report the number of observations, respectively. The bars display mean values from technical replicates, and whiskers show standard errors.

**FIGURE A4:**
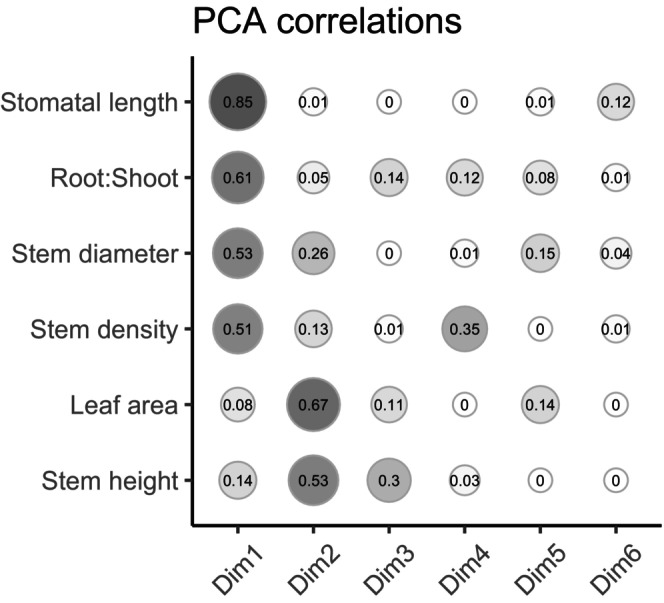
Correlations of plant functional traits (stomatal length, root‐to‐shoot ratio, stem diameter, stem density, leaf area, stem height) with PCA analysis dimensions (Dim.1–Dim.6) of three hybrid and three allopolyploid *Spartina* individuals tested in response to treatment combinations of drought versus well‐watered × elevated versus ambient CO_2_ level.

**FIGURE A6:**
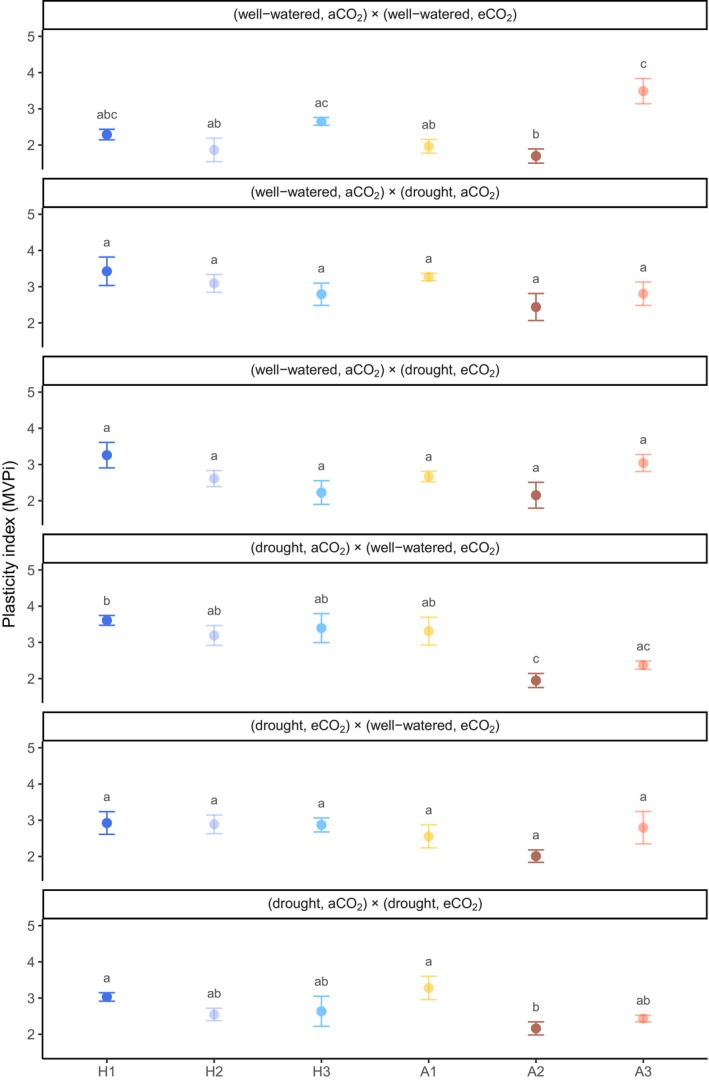
Multivariate plasticity index MVPi of three hybrid (H1–H3) and three allopolyploid (A1–A3) *Spartina* individuals in response to treatment combinations of drought vs. well‐watered × elevated (eCO_2_) versus ambient (aCO_2_) level. The letter‐codes (a, b, c) indicate differences between plant individuals (ANOVA followed by Tukey HSD post hoc tests, *p* < 0.05, df = 5; Means and error whiskers = standard error).

Phenotypic plasticity was intensively studied in the allopolyploid *Spartina anglica* (Thompson et al. 1991, 1991a, 1991b), demonstrating trait plasticity in reciprocal field transplantation and greenhouse experiments. However, comparisons between the hybrid and the allopolyploid that allow evaluating the effects of WGD separately from the effects of hybridization were not performed for functional traits as addressed in our study. Thompson et al. (1991b) identified two major forms of morphology in allopolyploid *Spartina* plants grown in different substrates in the greenhouse, categorized as “graceful” and “stocky” forms, which may reflect differences between the allopolyploid individuals in stem diameter and leaf area in our data. However, we cannot rule out legacy effects (cf. Hubbard 1969, 1970; Gray et al. 1991) as some individuals showed similar trait values in the greenhouse experiment as at the sampling site (e.g., in stem diameter and height; Table A1, Figure A3 D,E). Legacy effects point to an extensive body of literature demonstrating epigenetic and gene expression variation between closely related *Spartina* hybrids and allopolyploids (e.g., Salmon et al. 2005; Chelaifa et al. 2010; Cavé‐Radet et al. 2020; Giraud et al. 2021; Cavé‐Radet et al. 2023). The relation between legacy, genetic, and WGD‐mediated effects on plant traits and fitness remains to be investigated.

### 4.3 Impact of WGD on Plant Fitness and Biomass Allocation

Drought generally decreased biomass in both hybrid and allopolyploid *Spartina* cytotypes, and this decrease was found independent of CO_2_ levels. However, the decrease in biomass due to drought tended to be higher in the hybrid than in the allopolyploid (Figure 6C). Taking biomass as a proxy for fitness, the loss in fitness due to the combination of elevated CO_2_ levels and drought treatments was only slightly lower in the allopolyploid plants than in the hybrid plants, providing only weak support for our third hypothesis that WGD leads to increased plant fitness under the combination of these global change factors.

**FIGURE A7:**
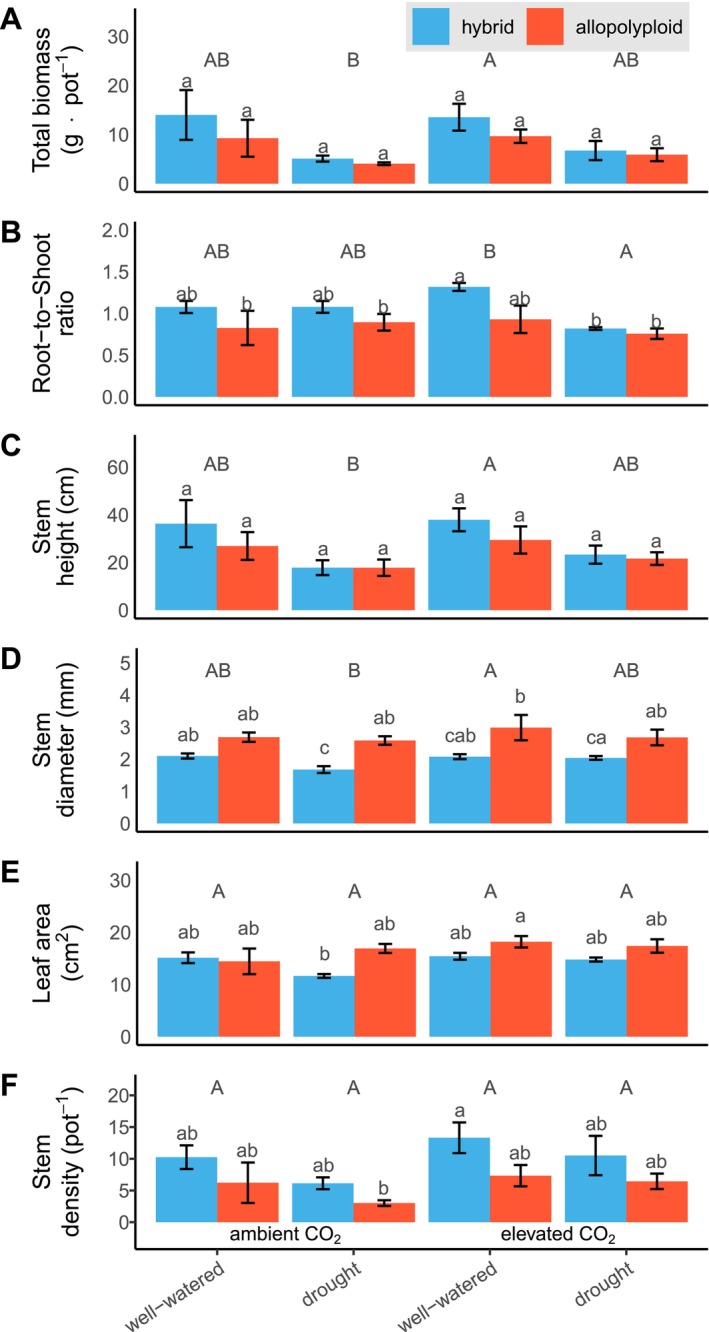
Total biomass (A), root‐to‐shoot ratio (B), stem height (C), stem diameter (D), leaf area (E), and stem density (F) of *Spartina* cytotypes (hybrid, allopolyploid) under drought (vs. well‐watered) and elevated CO_2_ (vs. ambient CO_2_) level treatments. The letter codes (A, B) indicate differences between treatments, while (a–e) denote differences between treatments and cytotypes (*p* < 0.05, *n* = 3; ANOVA followed by Tukey‐HSD post hoc test, respectively; Means and whiskers = standard error). For detailed statistics see Table A6.

Compared to the larger but less abundant leaves of the allopolyploid, the smaller yet more numerous leaves of the hybrid suggest similar light harvesting capacities among *Spartina* cytotypes (Figure A7E,F). Research on plant traits across different cytotypes in non‐*Spartina* taxa supports this hypothesis, indicating that leaf areas can increase and stem density can decrease following whole genome duplication (e.g., van Laere et al. 2011; Hao et al. 2013; Wang et al. 2017; Corneillie et al. 2019). This suggests compensation for WGD‐mediated effects on leaf area and stem density due to similar biomass production in the hybrid and allopolyploid descendant. However, the allocation of biomass in plants appeared to be different between the *Spartina* cytotypes under treatment with global change factors, with a more pronounced decrease in root‐to‐shoot ratio under drought and elevated CO_2_ in the hybrid (Figure 6B).

**FIGURE A8:**
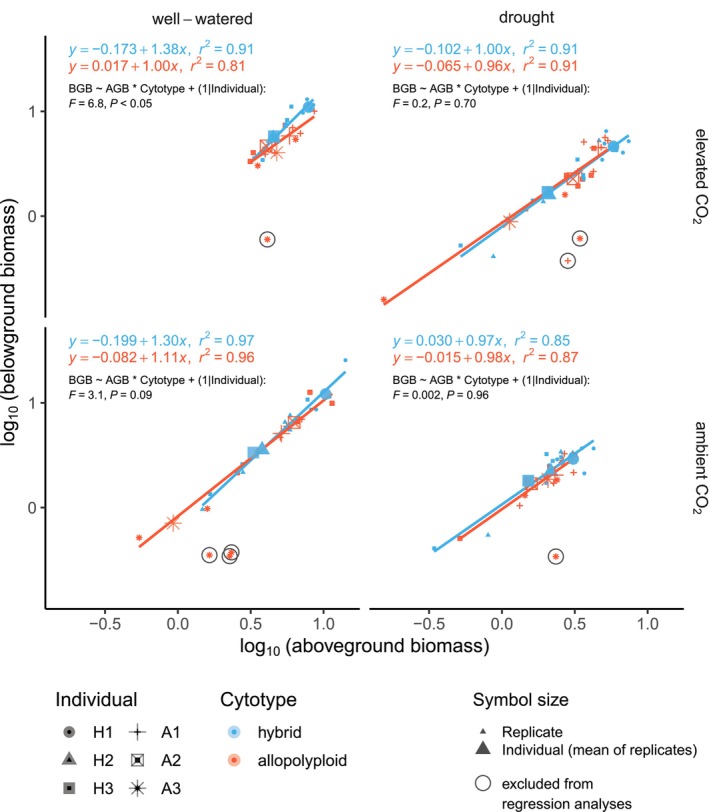
Allometric plots of aboveground (AGB) versus belowground (BGB) biomass (log_10_ transformed, respectively) for *Spartina*‐cytotypes (hybrid = H1–H3, allopolyploid = A1–A3) grown under two CO_2_ level (ambient, elevated) and different water availability treatments (ambient, drought). The regression analysis was conducted using the R‐function lm() and the resulting formulas (*y* = ax + b) including *r* ^2^ values are displayed. The effects of cytotypes were compared by means of a Linear Mixed Effects model using R‐function lmer(BGB ∼ AGB × Cytotype + (1|Individual)) with log_10_ transformed biomass. The model explains BGB increase by the fixed effects of AGB × Cytotype whereas individual replicates were introduced as random effects. The displayed *F* and *p* values show the statistics of the interaction term AGB × Cytotype, which was obtained from a Type III Analysis of Variance Table using Satterthwaite's method.

Different effects of drought and elevated CO_2_ levels on biomass allocation were reported in the literature, for example, drought mainly increases the fraction of root mass in herbaceous species (Poorter et al. 2012; Eziz et al. 2017), while elevated CO_2_ levels lead to increased aboveground biomass allocation in C_4_ species (Wand et al. 1999; Ainsworth and Long 2005; Domec et al. 2017; Paul et al. 2022; Koop‐Jakobsen and Dolch 2023). In line with Rogers et al. (1996) who concluded that crop type, resource supply, and experimental factors may explain a high variability in root‐to‐shoot ratio in response to elevated CO_2_ level, *Spartina* cytotypes differed particularly under elevated CO_2_ level in biomass allocation pattern (Figure 6B; Figure A8). This indicates that WGD can have strong effects on ecosystem functioning under future global change conditions, as both soil surface accretion and carbon accumulation in salt marshes are highly dependent on carbon input from plants (Hemminga et al. 1996; Kirwan and Megonigal 2013; Gorham et al. 2021; Temmink et al. 2022; Granse et al. 2024). Our data imply furthermore with respect to ecosystem functioning that WGD in *Spartina* induces shifts in the contributions from belowground biomass, supporting accretion and carbon sequestration, to those of aboveground biomass, including changes in biophysical plant‐environment interactions. The latter is attributed to the altered effects of aboveground plant traits on sediment trapping and tidal marsh hydrology, resulting from the less dense, but thicker, and likely sturdier stems of the allopolyploid. Particularly with regard to the invasiveness of *Spartina*, these differences may be important for management decisions because the sterile hybrid is discussed as a better choice for restoration projects due to its lower dispersal potential compared to the allopolyploid (Adsersen et al. 2010; Granse, Romeiro Motta, et al. 2022). We encourage further investigations to reveal differences between *Spartina* cytotypes.

### 4.4 Concluding Remarks

Hybridization and WGD pave the way not only for plant evolution but also for adaptation to climate change, entailing alterations in plant traits to maintain plant homeostasis. Our data indicate that WGD‐mediated persistent adjustment of plant trait expression is comparable to heritable variation and rapid evolution of plant traits (Vahsen, Blum, et al. 2023). Heritable variations in the allocation and distribution of belowground biomass in plants were demonstrated to correlate with the variation in salt marsh ecosystem attributes, namely marsh surface accretion in response to sea level rise (Kirwan and Megonigal 2013; Vahsen, Kleiner, et al. 2023). According to those studies, selection was directed towards plant traits that positively affect accretion, allowing plants to benefit from reduced flooding with seawater at higher elevations. Hybridization and WGD are common in the genus *Spartina* (Marchant 1967, 1977; Ainouche et al. 2012; Strong and Ayres 2013; Castillo et al. 2018) as well as in many other plant genera (Vallejo‐Marín et al. 2015; Barker et al. 2016; Čertner et al. 2017; Soltis et al. 2022), leading to variation in plant traits. Even though adaptive evolution in neopolyploids is limited by genetic bottlenecks, WGD induces ad‐hoc evolution in cell‐size‐related plant traits, accompanied by variations in plant traits at least at the individual level. This provides another source of variation for adaptation to global change.

## Author Contributions

**Dirk Granse:** conceptualization (lead), data curation (lead), investigation (lead), methodology (equal), visualization (lead), writing – original draft (lead). **Paul Wendt:** methodology (supporting). **Sigrid Suchrow:** methodology (equal), writing – review and editing (equal). **Dieter Hanelt:** supervision (equal), writing – review and editing (equal). **Jörg Fromm:** supervision (equal), writing – review and editing (equal). **Morgane Milin:** methodology (lead). **Oscar Lima:** methodology (lead). **Armel Salmon:** writing – review and editing (equal). **Malika Aïnouche:** writing – review and editing (equal). **Kai Jensen:** supervision (equal), writing – review and editing (equal).

## Conflicts of Interest

The authors declare no conflicts of interest.

## Figures and Tables

**FIGURE A5:**
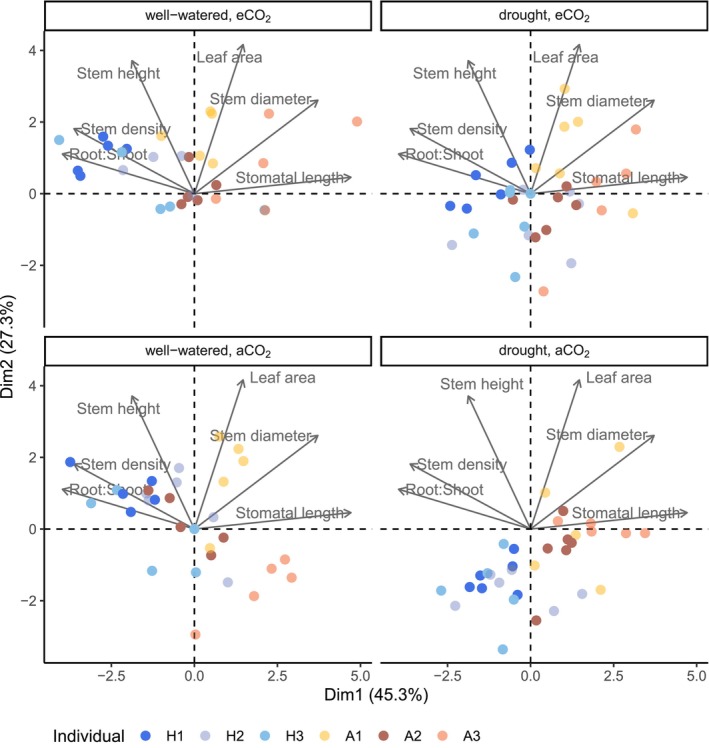
Multivariate trait‐scape (ordination plot) for PCA analysis of plant functional traits (root‐to‐shoot ratio, stem density, stem height, leaf area, stem diameter, stomatal length) of three hybrid (H1–H3) and three allopolyploid (A1–A3) *Spartina* individuals in response to treatment combinations of drought versus well‐watered × elevated (eCO_2_) versus ambient (aCO_2_) level (dots represent technical replicates of *Spartina* individuals).

## Data Availability

The research data will be published at PANGAEA—Database (Data Publisher for Earth & Environmental Science, Ecology; https://www.pangaea.de; Greenhouse experiment: Granse et al. 2024a; SSR marker results: Granse et al. 2024b).

## Appendix A

**TABLE A1:** Type of microhabitat, elevation, and plant traits during sampling in 2017 of *Spartina* donor individuals that were used as ramet donors for the greenhouse experiment. Stem diameter and height are reported as mean of three measurements in the field, respectively.

Sample	Cytotype	Habitat	Elevation MHT [cm]	Stem density [m^−2^]	Stem diameter [mm]	Stem height [cm]
H1	Hybrid	Tidal flat hummock	−86	1329	1.5	35
H2	Hybrid	Tidal flat hummock	−62	4933	1.6	12
H3	Hybrid	Pioneer marsh	−6	2304	2.0	37
A1	Allopolyploid	Pioneer marsh	23	620	4.3	90
A2	Allopolyploid	Low/high marsh	45	384	3.8	40
A3	Allopolyploid	Tidal creek	−10	443	5.0	56

**TABLE A2:** Microsatellite markers developed from *Spartina maritima* genomic data (AS, amplicon size in bp) published by Castillo et al. (2018).

Locus	Seq. forward (5′ −> 3′)	Seq. reward (5′ −> 3′)	AS	SSR
MS02	ATATTCCGATCCCCTCCTTG	TTCGATCGGTCATGTTTTGA	265	(AAAG)n
MS07	CAGAATCACCATCATCAGCG	TTCCATTTTTCAGGGTGAGC	275	(TGGCAG)n
MS13	CTTGACCGCAACCAGTATGA	CCCAGGGCAATGGTTATACA	262	(TTCT)n
MS14	TGAGTTTGAGTTCACGGTTCA	ATGTGATGCCATTTCCACAA	271	(AAAG)n
MS15	TGCATTGCAGCAAGAGAATC	CGCTAGCTGATCCTGGAAAC	264	(GATG)n
MS16	GGGACACGGGATAGGAAAGT	CCGCCGTGCAATTATTTATC	259	(GTGGA)n
MS17	TTTGTTCAGCTTCAGCATGG	TTCTTGCAGTCGTTCTGTGC	273	(GAAA)n
MS18	TCTTATGGACCCCTTGCAGT	CATCCGATTGGCGTAAGATT	260	(TGATA)n

**TABLE A3:** PCR solution.

Solution	Volume
5× Green GoTaq Flexi Buffer	3.0 μL
MgCl_2_	1.2 μL
dNTPs	0.3 μL
GoTaq Flexi polymerase	0.06 μL
Primer DNA (F, R)	0.1 μL
Sample DNA	0.6 μL

**TABLE A4:** PCR program.

Step	Temperature	Duration	Cycles
Initial DNA			
Denaturation	94°C	120 s	
Denaturation	94°C	30 s	|
Annealing	50°C	30 s	| × 35
Extension	72°C	90 s	|
Final extension	72°C	420 s	

**TABLE A5:** *Spartina* genotypes, multilocus genotypes and their frequency for homoploid F_1_‐hybrid (individuals: H1–H3) and its allopolyploid descendant (individuals: A1–A3). Alleles were scored as present = 1 or absent = 0 for the eight microsatellite loci (MS02, MS07, MS13–MS18). The number of allelic differences (missing or additional alleles) between genotypes (*B*–*R*) and the most prominent genotype *A* (16 of 25 alleles) is denoted (Alleles changed) with a minus sign (−) for missing alleles and a plus sign (+) for additional alleles.

Genotype	Multilocus genotype	Alleles	Frequency (%, *n*)		
			Hybrid	Allopolyploid	Total
		Changed	(*n* = 7)	(*n* = 72)	(*n* = 79)
*A* (A3)	0111011001101111111100100	−0/+0	42.9 (3)	56.9 (41)	55.7 (44)
*B*	111101100110111111110000	−0/+1	0.0 (0)	8.3 (6)	7.6 (6)
*C* (A2)	0111011001101111111101100	−0/+1	0.0 (0)	6.9 (5)	6.3 (5)
*D* (H2)	0111111001101111111100100	−0/+1	14.3 (1)	2.8 (2)	3.8 (3)
*E*	0111011001101111111100110	−0/+1	0.0 (0)	2.8 (2)	2.5 (2)
*F*	0111011101101111111100100	−0/+1	0.0 (0)	1.4 (1)	1.3 (1)
*G*	0111011011101111111100100	−0/+1	0.0 (0)	1.4 (1)	1.3 (1)
*H* (A1)	1111111001101111111100100	−0/+2	0.0 (0)	1.4 (1)	1.3 (1)
*I*	1111111101101111111101100	−0/+4	0.0 (0)	1.4 (1)	1.3 (1)
*J* (H1)	0111011001101011111100100	−1/+0	14.3 (1)	5.6 (4)	6.3 (5)
*K*	0111011001101111111000100	−1/+0	0.0 (0)	2.8 (2)	2.5 (2)
*L* (H3)	0110011001101111111100100	−1/+0	14.3 (1)	1.4 (1)	2.5 (2)
*M*	0111010001101111111100100	−1/+0	0.0 (0)	1.4 (1)	1.3 (1)
*N*	0111011001111101111100100	−1/+1	0.0 (0)	1.4 (1)	1.3 (1)
*O*	0111011001101011111110100	−1/+1	0.0 (0)	1.4 (1)	1.3 (1)
*P*	1111011001101111111001100	−1/+2	0.0 (0)	1.4 (1)	1.3 (1)
*Q*	0111011001101111111110001	−1/+2	0.0 (0)	1.4 (1)	1.3 (1)
*R*	0111011001001001111100100	−3/+0	14.3 (1)	0.0 (0)	1.3 (1)

**TABLE A6:** ANOVA results of *Spartina* cytotype (hybrid, allopolyploid) responses (stomatal length, root‐to‐shoot ratio, and total biomass) to different CO_2_ levels (_treat_CO_2_: ambient, elevated CO_2_) and water availability (_treat_H_2_O: well‐watered, drought); in bold font: *p* < 0.05.

	Df	Stomatal length			Root‐to‐shoot ratio			Total biomass		
		SSq	*F*	*p*	SSq	*F*	*p*	SSq	*F*	*p*
Cytotype	1	0.559	94.4	< **0.001**	0.262	10.0	**0.006**	0.703	2.2	0.15
_treat_H_2_O	1	0.004	0.7	0.41	0.172	6.5	**0.021**	3.613	11.5	**0.004**
_treat_CO_2_	1	0.001	0.1	0.74	0.000	0.0	0.97	0.356	1.1	0.30
_treat_H_2_O × _treat_CO_2_	1	0.000	0.0	0.99	0.200	7.6	**0.014**	0.021	0.1	0.80
Cytotype × _treat_H_2_O	1	0.007	1.2	0.29	0.058	2.2	0.16	0.156	0.5	0.49
Cytotype × _treat_CO_2_	1	0.006	1.0	0.33	0.002	0.1	0.77	0.054	0.2	0.68
Cytotype × _treat_H_2_O × _treat_CO_2_	1	0.001	0.2	0.67	0.043	1.7	0.22	0.009	0.0	0.87
Residuals	16	0.095			0.420			5.023		

	Df	Stem height			Stem diameter			Leaf area			Stem density		
		SSq	*F*	*p*	*SSq*	*F*	*p*	SSq	*F*	*p*	SSq	*F*	*p*
Cytotype	1	0.161	1.2	0.29	0.062	54.1	< **0.001**	417.796	9.2	**0.008**	13.416	8.9	**0.009**
_treat_H_2_O	1	1.475	11.2	**0.004**	0.007	6.1	**0.026**	24.460	0.5	0.47	4.660	3.1	0.10
_treat_CO_2_	1	0.209	1.6	0.23	0.004	3.7	0.07	222.101	4.9	**0.041**	6.890	4.5	**0.049**
_treat_H_2_O × _treat_CO_2_	1	0.037	0.3	0.60	0.002	2.0	0.18	1.043	0.0	0.88	0.607	0.4	0.54
Cytotype × _treat_H_2_O	1	0.094	0.7	0.41	0.002	1.6	0.22	118.887	2.6	0.12	0.240	0.2	0.70
Cytotype × _treat_CO_2_	1	0.001	0.0	0.92	0.001	1.2	0.30	4.166	0.1	0.77	0.002	0.0	0.97
Cytotype × _treat_H_2_O × _treat_CO_2_	1	0.001	0.0	0.94	0.004	3.5	0.08	138.387	3.1	0.10	0.037	0.0	0.88
Residuals	16	2.117			0.018			723.008			24.252		
